# The Effects of Adjuvant Therapies on Embryo Transfer Success

**Published:** 2017

**Authors:** Rachael Shirlow, Martin Healey, Michelle Volovsky, Vivien MacLachlan, Beverley Vollenhoven

**Affiliations:** 1- Monash University, Melbourne, Australia; 2- Monash Health, Melbourne, Australia; 3- University of Melbourne, Melbourne, Australia; 4- Royal Women’s Hospital, Melbourne, Australia; 5- Monash IVF, Melbourne, Australia; 6- Royal Melbourne Hospital, Melbourne, Australia

**Keywords:** Adjuvant drug therapy, Aspirin, Melatonin, Steroids

## Abstract

**Background:**

Many adjuvant therapies are employed during IVF treatment in an attempt to improve outcomes. The objective of our study was to evaluate the impact of thirteen adjuvants (Intralipid, steroids, melatonin, coenzyme Q10, Filgrastim, testosterone, DHEA, growth hormone, antibiotics, hCG infusion, aspirin, enoxaparin/heparin and dopamine agonists) on the success of embryo transfers.

**Methods:**

This is a retrospective cohort study of all embryo transfers between January 2010 and April 2015 from a multi-site IVF clinic. To ensure data independence, random number was applied to each included transfer and used to pick an individual transfer for each patient (n=13,372). Outcomes were clinical pregnancy, live birth and pregnancy loss. Univariate comparison with Chi square testing and logistic regression analysis were used. The level of significance was p<0.05.

**Results:**

Steroid use was significantly associated with both reduced clinical pregnancy loss (aOR 0.39, CI 0.19–0.76) and improved live birth rates (aOR 1.40, CI 1.11–1.77). While aspirin was associated with improved live birth rates (aOR 1.48, CI 1.08–2.02), melatonin was linked with reduced rates (aOR 0.66, CI 0.45–0.96). Analyses for all other adjuvant therapies did not reach statistical significance after logistic regression.

**Conclusion:**

Many of the interventions investigated in this study fail to significantly demonstrate any effects on the success of embryo transfers. Our analysis results show negative effects with the use of melatonin; however, use of aspirin or steroids demonstrated promising, potentially beneficial outcomes. Additional exploration is needed to guide evidence-based practice.

## Introduction

With up to one in six couples affected by infertility (1), many turn to *in vitro* fertilization (IVF) to overcome various barriers to conception. Yet, continuous failure to achieve a pregnancy despite optimization of IVF processes is extremely distressing for couples and presents a significant challenge to clinicians.

IVF protocols can be targeted to overcome individualized fertility difficulties. Areas open to manipulation include ovarian stimulation, oocyte collection and fertilization, with the final stage in the process as embryo transfer. The success of embryo transfer is determined largely by embryo quality and endometrial receptivity (2–4). Optimal folliculogenesis and oocyte maturation are essential to oocyte quality with a flow-on effect to embryo quality. Therefore, therapies proposing to improve the follicular micro-environment for these processes are currently being explored. Furthermore, two-thirds of implantation failures are suspected to be due to poor endometrial receptivity (3). Some evidence suggests an immunological role for reduced endometrial receptivity (5–7) and it is for this reason that many interventions targeted at achieving an “ideal” immune environment are also being investigated.

There are many adjuvant therapies to IVF with a wide variety of different mechanisms of action. This study examined thirteen adjuvants to IVF and attempted to reduce uncertainty surrounding their use. These therapies have been proposed to improve the success of embryo transfer by enhancing embryo quality or implantation through follicular development, oocyte maturation and/or endometrial receptivity.

Intralipid is a fat emulsion constituting soya bean oil, egg phospholipids and glycerine that has been shown to have immunosuppressive properties (8–9). Glucocorticoids have also been found to have immune-regulatory properties and alter natural killer cell activity (10). The antioxidant, melatonin, is involved in reproductive processes including follicular development, oocyte maturation and ovulation (11). Co-enzyme Q10, also holding antioxidant properties, is important for energy metabolism and preventing oxidative damage to cell membranes (12). Filgrastim is a granulocyte colony-stimulating factor (G-CSF) analogue and is employed in IVF because natural G-CSF is suspected to have an important role in oocyte maturation, the ovulation process and endometrial receptivity (13). As androgens, testosterone and DHEA have importance in the early stages of oocyte growth as well as oocyte quality (14). Growth hormone modulates the effect of FSH on granulosa cells through up-regulation of the synthesis of insulin-like growth factor 1, which is important in follicular development and oocyte maturation (15). Antibiotics have been proposed to improve endometrial receptivity by reducing negative impacts of microbial colonization (16). Human chorionic gonadotropin (hCG) promotes immunological tolerance and angiogenesis that assists with embryo implantation (17). Aspirin has been recognized to have anti-inflammatory and anti-platelet properties and has been proposed for use in reproductive medicine with the idea that it may enhance uterine perfusion and improve endometrial receptivity (18, 19). Blood thinning agents, enoxaparin and heparin, may have additional effects on embryo implantation and invasion of the endometrium (20). And lastly, dopamine agonists are largely used as an adjuvant to IVF treatment for their effectiveness in reducing ovarian hyperstimulation syndrome but their effects on the follicular fluid micro-environment may also affect implantation, pregnancy and live birth rates (21).

These adjuvant therapies understandably garner attention from patients and clinicians alike; however, these treatments often have limited practical evidence and little is known about their effects on embryo transfer and the subsequent pregnancy and live birth rates. Such therapies may have theoretical potential and would have significant impacts on IVF outcomes if proven beneficial, yet they remain controversial because of the limited evidence for their efficacy.

This study examined a cohort of patients at the stage of embryo transfer and focused on one transfer per patient. The study aimed to evaluate the impact of thirteen adjuvant therapies (Intra-lipid, steroids, melatonin, coenzyme Q10, Filgrastim, testosterone, DHEA, growth hormone, antibiotics, hCG infusion, aspirin, enoxaparin/heparin and dopamine agonists) on the success of embryo transfer, including the clinical pregnancy and live birth rates.

## Methods

A retrospective cohort study was performed based on the standardized database from a private multi-site IVF clinic. From a total of 115,033 embryo transfers, those occurring between January 2010 and April 2015 were collected (n=45,455) as this covered the period when adjuvant usage was the greatest. Where information was missing or other adjuvants were used and case numbers were less than 20, cycles were excluded (n=9,663) leaving 35,792 embryo transfers. To ensure data independence, a random number was applied to each transfer and used to pick an individual transfer for each patient (n=13,372) deleting the remaining 22,420 duplicate cycles. The inclusions and exclusions of embryo transfers for this study are shown as a flowchart in figure 1.

Stimulation was achieved either by downregulation of gonadotropin stimulation or gonadotrophin antagonist stimulation with or without pre-treatment with the oral contraceptive pill for timing. Embryo freezing was accomplished by vitrification. Adjuvant protocols are outlined in table 1.

**Table 1.** Adjuvant protocols

**Adjuvant**	**Protocol**
**Antibiotics**	Doxycycline 100 *mg* for 4 days beginning from vaginal egg pick up (VPU) [or] Azithromycin 1g given on the night before VPU [or] Augmentin duo forte BD for 4–5 days post-VPU
**Aspirin**	100 *mg* from VPU until pregnancy test
**Enoxaparin / Heparin**	20–40 *mg* from VPU until pregnancy test
**Steroids**	Various protocols 10–30 *mg* daily Used from stimulation until - Some stopping at pregnancy test - Some stopping until the 1st trimester
**Melatonin**	4 *mg* given at night Variable start time, but between 2 days and up to 8 weeks before egg collection Ceased on egg collection
**Dopamine agonist**	0.5 *mg* cabergoline orally daily From day of hCG or egg pick up and continuing to 5 days or until pregnancy test [or] Bromocriptine 7.5 *mg* oral or PV daily starting with hCG trigger until pregnancy test
**HCG infusion**	Dilution of 1500 *IU* of hCG in 125 microlitres of blastocyst media, 40 microlitres of this was infused into the uterus 10 *min* to immediately prior to embryo transfer
**DHEA**	75 *mg* daily Given for 3 *mths* prior to cycle
**Testosterone**	Given as pre-treatment between 5–21 days before the start of a cycle Given as either a 2.5 *mg* patch or 10 *mg* topical gel
**Growth hormone**	12 *IU/day* from day of FSH until trigger injection Intramuscular injection
**Intralipid infusion**	200 *mls* of Intralipid 20% was given intravenously over two hours 7–10 days before embryo transfer. When a pregnancy was confirmed, additional doses were administered at 7, 9, 11 and 13 weeks
**Filgrastim**	Intrauterine Given 2 days before embryo transfer 300 *μg* into uterus 2 days before ET
**Co-enzyme Q10**	150–300 *mg* given at night Variable start time, but between 2 days and up to 8 weeks before egg collection Ceased on egg collection

### Statistical analysis:

Proportions were compared using Chi-square test with Mantel-Haenszel correction or Fisher exact 2 tailed test if there was a value of <5, and crude odds ratios calculated. Continuous variables were compared with the Mann Whitney-U test. Logistic regression was used to produce adjusted odds ratios. The following variables were used in logistic regression model as potential covariates: number of previous treatment cycles; body mass index; embryo age at transfer; number of embryos transferred; fertilization (standard *vs*. ICSI); transfer (fresh vs. frozen); woman’s age at egg collection; clinic site (central *vs*. satellite vs. interstate); smoking status; diabetic status; total number of previous pregnancies or deliveries; number of consecutive embryo transfers since a chemical pregnancy or live birth; etiological factors for infertility (endometriosis, fibroids, PCOS, ovulation dysfunction, tubal factors, male factor, idiopathic and unknown); ultrasound findings (PCO, endometrioma); adjuvants (antibiotics, aspirin, steroids, melatonin, prolactin antagonists, HCG infusion, DHEA, testosterone, growth hormone, Intralipid and Filgrastim, co-enzyme Q10). Adjuvants with 20 or fewer cases were excluded from the logistic regression analysis (IVIG, sildenafil, pentoxifylline, stem cells). Statistical significance was set at p<0.05.

This study had Human Research Ethics Committee’s approval.

## Results

Of the 13,372 unique embryo transfer cycles, there were 1,904 where one or more adjuvants were used. A comparison of the demographics and cycle characteristics for those with and without adjuvants is seen in table 2. This shows that women receiving adjuvant therapies are a distinct group who are older, have had more prior IVF cycles, are more likely to be having a double embryo transfer, have had a Day 3 embryo transfer and a fresh cycle. The number of adjuvants used was: one (n=913), two (n=536), three (n=239), four (n=81), five (n=51), six (n=41), seven (n=33), eight (n=9) and nine (n=1). The following adjuvants were used and recorded: antibiotics (n=274), aspirin (n=431), enoxaparin/heparin (n=834), steroids (n=997), melatonin (n=341), prolactin antagonist (n=58), HCG infusion (n=189), DHEA (n= 75), testosterone (n=82), GH (n=120), Intralipid (n=311), Filgrastim (n=102) and Co-enzyme Q10 (n=25).

**Table 2.** Comparison of demographics and cycle features (median 95%CI)

**Feature**	**≥1 Adjuvant (n=1,904)**	**No adjuvants (n=11,468)**	**Crude OR**	**Chi square p**
**Number of previous IVF cycles**	4 (1–16)	2 (1–11)		<0.001
**BMI**	23.7 (18.2–38.7)	23.8 (18.3–39.3)		0.17
**Embryo age (days)**	D2-7.1% (136) D3-29.8% (568) D4-3.2% (60) D5/6/7-59.9% (1139)	D2-5.6% (639) D3-20.5% (2355) D4-2.7% (309) D5/6/7-70.6% (8099)		<0.001
**Number of 2 embryo transfers**	26.3% (501/1904)	12.8% (1464/10004)	2.44 (2.17–2.74)	<0.001
**Insemination by ICSI**	83.3% (1586/1904)	72.1% (8267/11468)	1.93 (1.70–2.20)	<0.001
**Frozen ET**	34.0% (648/1904)	39.6% (4543/11468)	0.79 (0.71–0.87)	<0.001
**Woman’s age at egg collection (years)**	38.5 (28.1–45.0)	35.6 (25.9–44.9)		<0.001
**Number of previous pregnancies**	1 (0–5)	1 (0–4)		0.016
**Number of previous deliveries**	0 (0–2)	0 (0–2)		<0.001
**Number of consecutive ETs without chemical pregnancy**	1 (0–9)	1 (0–6)		<0.001
**Number of consecutive ETs without clinical pregnancy**	1 (0–9)	1 (0–6)		<0.001
**Number of consecutive ETs without live birth**	2 (0–12)	1 (0–7)		<0.001
**Site – central**	90.7% (1727/1904)	62.1% (7121/11468)	5.96 (5.06–7.01)	<0.001
**Site – satellite**	3.9% (74/1904)	10.9% (1248/11468)	0.33 (0.26–0.42)	<0.001
**Site - interstate**	5.4% (103/1904)	27.0% (3099/11468)	0.15 (0.13–0.19)	<0.001
**Smoker**	1.9% (37/1904)	3.0% (344/11468)	0.64 (0.45–0.91)	0.01
**Year of ET**	2012 (2010–2015)	2012 (2010–2015)		<0.001
**Diabetic**	1.1% (20/1904)	0.9% (107/11468)	1.13 (0.68–1.86)	0.62
**Etiologies**				
**Male factor**	0.1% (1/1904)	0.02% (2/11468)	3.01 (^**^–^**^)	0.37
**Tubal factor**	8.2% (157/1904)	10.1% (1164/11468)	0.80 (0.67–0.95)	0.01
**Endometriosis**	11.7% (222/1904)	11.0% (1264/11468)	1.07 (0.91–1.24)	0.41
**Fibroids**	3.7% (71/1904)	2.8% (319/11468)	1.35 (1.03–1.77)	0.02
**Ovarian dysfunction**	6.2% (118/1904)	6.9% (787/11468)	0.90 (0.73–1.10)	0.28
**PCOS**	3.0% (58/1904)	6.1% (703/11468)	0.48 (0.36–0.64)	<0.001
**Idiopathic**	23.2% (442/1904)	26.8% (3075/11468)	0.83 (0.74–0.93)	<0.001
**Unknown**	31.0% (590/1904)	26.8% (3071/11468)	1.23 (1.10–1.37)	<0.001
**Ultrasound - endometrioma**	0.3% (5/1904)	0.4% (44/11468)	0.68 (0.24–1.80)	0.42
**Ultrasound - PCO**	15.7% (298/1904)	18.8% (2160/11468)	0.80 (0.70–0.91)	<0.001

** Confidence limits invalid

Comparison of clinical pregnancy rates and live birth rates between women with and without specific adjuvants is shown in tables 3 and 4, respectively. On comparison of clinical pregnancy loss rates, the only adjuvant therapy to demonstrate significant findings was steroids. Use of steroids gave a crude non-significant odds ratio of 0.90 (0.64–1.25). However, once adjusted for logistic regression, a statistically significant reduction to clinical pregnancy loss rates was seen with an adjusted odds ratio of 0.39 (0.19–0.76, p=0.006). Analyses of clinical pregnancy loss rates for all other adjuvant therapies were non-significant on univariate and multivariate testing.

**Table 3.** Clinical pregnancy rates with adjuvants

	**Use of one adjuvant only**	**No adjuvant used**	**Crude OR**	**Adjusted OR**	**p**
**Antibiotics**	36.5% (100/274)	39.3% (5144/13098)	0.89 (0.69–1.15)	1.07 (0.77–1.49)	0.71
**Aspirin**	36.9% (159/431)	39.3% (5085/12941)	0.90 (0.74–1.11)	1.27 (0.98–1.64)	0.07
**Enoxaparin/heparin**	33.2% (277/834)	39.6% (4967/12538)	0.76 (0.65–0.88)	0.89 (0.68–1.17)	0.41
**Steroids**	33.5% (334/997)	39.7% (4910/12375)	0.77 (0.67–0.88)	1.07 (0.88–1.30)	0.51
**Melatonin**	27.9% (95/341)	39.5% (5149/13031)	0.59 (0.46–0.76)	0.87 (0.61–1.25)	0.46
**Dopamine Ag**	53.4% (31/58)	39.2% (5213/13314)	1.78 (1.03–3.08)	1.13 (0.56–2.25)	0.74
**HCG infusion**	30.2% (57/189)	39.3% (5187/13183)	0.67 (0.48–0.92)	0.78 (0.51–1.20)	0.26
**DHEA**	29.3% (22/75)	39.3% (5222/13297)	0.64 (0.38–1.08)	1.12 (0.59–2.11)	0.74
**Testosterone**	13.4% (11/82)	39.4% (5233/13290)	0.24 (0.12–0.46)	0.44 (0.18–1.01)	0.05
**Growth hormone**	20.8% (25/120)	39.4% (5219/13252)	0.41 (0.25–0.64)	0.57 (0.32–1.01)	0.05
**Intralipid infusion**	32.2% (100/311)	39.4% (5144/13061)	0.73 (0.57–0.93)	1.02 (0.68–1.54)	0.92
**Filgrastim**	21.6% (22/102)	39.4% (5222/13270)	0.42 (0.26–0.69)	0.89 (0.42–1.88)	0.76
**Co-enzyme Q10**	12.0% (3/25)	39.3% (5241/13347)	0.21 (0.05–0.74)	0.83 (0.42–1.87)	0.79

**Table 4.** Live birth rates with adjuvants

	**Use of one adjuvant only**	**No adjuvant used**	**Crude OR**	**Adjusted OR**	**p**
**Antibiotics**	31.8% (87/274)	33.5% (4385/13098)	0.92 (0.71–1.20)	1.11 (0.75–1.64)	0.59
**Aspirin**	31.3% (135/431)	33.5% (4337/12941)	0.90 (0.73–1.08)	1.48 (1.08–2.02)	0.014
**Enoxaparin/heparin**	28.7% (239/834)	33.8% (4233/12538)	0.79 (0.67–0.92)	1.05 (0.76–1.45)	0.76
**Steroids**	29.0% (289/997)	33.8% (4183/12375)	1.05 (0.91–1.21)	1.40 (1.11–1.77)	0.004
**Melatonin**	23.8% (81/341)	33.7% (4391/13031)	0.61 (0.47–0.79)	0.66 (0.45–0.96)	0.032
**Dopamine Ag**	53.4% (31/58)	33.4% (4441/13314)	2.29 (1.33–3.96)	1.58 (0.74–3.39)	0.24
**HCG infusion**	25.9% (49/189)	33.6% (4423/13183)	0.69 (0.49–0.97)	0.74 (0.43–1.25)	0.26
**DHEA**	22.7% (17/75)	33.5% (4455/13297)	0.58 (0.33–1.03)	1.09 (0.51–2.35)	0.82
**Testosterone**	11.0% (9/82)	33.6% (4463/13290)	0.24 (0.11–0.50)	0.54 (0.20–1.46)	0.23
**Growth hormone**	19.2% (23/120)	33.6% (4449/13252)	0.47 (0.29–0.75)	0.81 (0.41–1.61)	0.55
**Intralipid infusion**	28.9% (90/311)	33.6% (4382/13061)	0.81 (0.62–1.04)	0.98 (0.61–1.59)	0.98
**Filgrastim**	20.6% (21/102)	33.5% (4451/13270)	0.51 (0.31–0.85)	1.00 (0.41–2.41)	1.00
**Co-enzyme Q10**	8.0% (2/25)	33.5% (4470/13347)	0.17 (0.03–0.75)	1.22 (0.22–6.65)	0.82

Use of steroids was associated with both reduced clinical pregnancy loss and improved live birth rates. While aspirin was associated with improved live birth rates, melatonin was linked with reduced rates.

## Discussion

Many of the adjuvant therapies examined have theoretical potential benefits yet our study demonstrates that numerous interventions fail to demonstrate any statistically significant improvements to embryo transfer success.

Unsurprisingly with our population, negative effects are seen on the univariate analyses of a range of therapies. As seen from the demographics of table 2, the women using adjuvant therapies are often those who have previously been unsuccessful at IVF, seldom achieving optimal embryo age targets and/or opportunity for embryo freezing. After allowing for confounders and controlling for obvious differences between our cases and controls with logistic regression, these negative effects became non-significant for all therapies except melatonin.

The negative impact on embryo transfers seen with the use of melatonin in our study was surprising. Our analysis is the first to report a statistically significant reduction in live birth rates. Theoretically, melatonin has promising uses within the IVF cycle. Studies have demonstrated high concentrations of melatonin in pre-ovulatory follicular fluid (11) as well as melatonin receptors on granulosa cells (22). Oxidative stress is a possible cause of poor oocyte quality and decreased fertilization rates and thus melatonin, with its known anti-oxidant abilities and proven safety, has been employed as an adjuvant therapy for its potential benefits (22–23). Studies have presented significant positive relation to its efficacy in improving oocyte quality but there is limited data which specifically assessed its effect on pregnancy outcomes. Literature that has assessed pregnancy outcomes has reported conflicting findings on melatonin’s efficacy in IVF, but results have been either non-significant or positive. Showell’s Cochrane review (24) of antioxidant use found no association of increased pregnancy rates in women receiving melatonin in the two randomized controlled trials it included. The included trials involved a total of 145 patients of which 70 received melatonin. Our study measured melatonin’s effects on embryo transfer and implantation success and our numbers are almost five-fold those demonstrated in Showell’s Cochrane review as 341 patients in our study received melatonin. Although not randomized, our larger sample size provides the opportunity to demonstrate a difference that perhaps smaller randomized controlled trials would not. The negative impact demonstrated in our results is dissimilar to the existing knowledge possibly because melatonin’s theoretical benefits have earlier effect within the IVF cycle. It is important that the negative effect demonstrated on implantation and subsequent live birth rates is investigated fully and the use of melatonin in IVF is reassessed in high quality study designs with adequate sample sizes.

Our analysis of testosterone use did not demonstrate significant impacts upon embryo transfers. Our results align with Nagel et al.’s 2015 Cochrane review (25) which concluded that, once adjusted to reduce performance bias, there were no statistically significant differences with the use of testosterone. However, Nagel’s unadjusted data appeared promising whereas our study’s univariate analysis suggests that testosterone negatively affects pregnancy and live birth rates. Our results possibly deviate from Nagel’s findings as his review included studies examining the whole IVF cycle, in contrast to our focus on embryo transfer success. However, the theoretical benefits of testosterone and other androgens are proposed to include modulation of the decidualization process and decidual-trophoblast interactions, which are regarded as “the critical processes that control embryo implantation” (26), so the negative shift demonstrated by testosterone within our study is surprising and warrants further exploration with high-quality studies.

The use of growth hormone within our study similarly showed negative impacts on embryo transfer success on univariate data but did not reach significance when confounders were controlled for. To date, there have only been small-sample studies examining the effects of growth hormone use in IVF but findings have been either non-significant or promising (27). The proposed use of growth hormone as an adjuvant therapy in IVF comes from studies illustrating its modulation of FSH effects on granulosa cells through up-regulation of the synthesis of insulin-like growth factor 1 (15). These processes are important in follicular development and oocyte maturation and thus an improved oocyte quality was hypothesized to lead to increased embryo transfer success. With our focus on success following embryo transfer, our univariate results contradicted this and instead suggested that endometrial receptivity may be adversely affected by growth hormone, despite its potential beneficial effects on earlier processes within the IVF cycle such as oocyte quality and production.

No significant differences were found in embryo transfer outcomes with the use of an hCG infusion. hCG promotes immunological tolerance of the embryo and may have positive effects on implantation through various mechanisms including angiogenesis, increased endometrial cell receptivity and a reduction of natural killer cells (17). Our data on hCG has previously been published (28); however, results differ slightly due to the difference in the study design. The efficacy of hCG infusions as an adjuvant to IVF therapy have revealed conflicting results, but similar to Craciunas et al.’s Cochrane review (29), our findings do not support its use in IVF cycles as no significant positive effect can be demonstrated.

Use of co-enzyme Q10 did not significantly impact on the success of embryo transfers within our study. Studies have suggested that coenzyme Q10 supplementation has positive effects on oocyte quality by improving mitochondrial performance, scavenging free radicals and preventing oxidative damage (30–31). Within an aged animal model, Ben-Meir et al. (32) demonstrated that coenzyme Q10 supplementation “delayed depletion of ovarian reserve, restored oocyte mitochondrial gene expression and improved mitochondrial activity”. Furthermore, Turi et al.’s study (12) was the first to demonstrate the presence of coenzyme Q10 in follicular fluid. For these reasons, it has been theorized to improve implantation rates. This theory could not be supported or rejected significantly in our study.

Filgrastim is currently utilized during IVF in an attempt to improve endometrial receptivity. Filgrastim is a G-CSF analogue and natural G-CSF receptor expression has been demonstrated in a wide variety of tissue types, including reproductive organs (13). The use of Filgrastim in IVF, however, has revealed conflicting results from studies of varying quality and populations (13, 33–35). The latest data, Aleyasin et al.’s 2016 recent randomized controlled trial (33), has demonstrated significantly higher implantation rates and chemical and clinical pregnancy rates with the use of Filgrastim using a protocol of 300 *mcg* administered subcutaneously one hour before embryo transfer. Our study did not support these findings with the use of Filgrastim administered as a 300 *mcg* intrauterine infusion two days before embryo transfer. Similarly, Barad et al.’s randomized controlled trial (34) found no statistical significance with the use of the same intrauterine Filgrastim dose given five days before embryo transfer. Aleyasin’s findings (33) may suggest a role for Filgrastim within IVF cycles prior to transfer and warrant further exploration of alternate methods of administration.

There is a varying degree of evidence for the use of enoxaparin or heparin in IVF, but research thus far has been promising or non-specific (36). Little investigation has been made into the potential effects of enoxaparin, but heparin is proposed to have importance in the adhesion of the blastocyst to the endometrium and subsequent invasion (20). Our results align with current data in demonstrating no obvious benefit with the use of enoxaparin/heparin.

Our analysis of Intralipid use in IVF reported no difference in outcomes once confounding factors have been allowed for. Intralipid has been suggested for overcoming poor uterine receptivity due to studies demonstrating its ability to suppress natural killer cell activity (8–9). Associations have been made between abnormal natural killer cell activity and recurrent implantation failure (5–7) and hence it was thought that having an immunological approach could greatly impact the success of embryo transfers. However, the clinical significance of natural killer cells in implantation continues to be debated and indeed our findings do not demonstrate any significant effects. On univariate analysis, our results instead seem to align with the thinking that natural killer cells may have a protective, beneficial effect on reproductive outcomes and that their suppression could be harmful (37).

The use of antibiotics and DHEA failed to demonstrate any significant effects before or after logistic regression.

Antibiotics have been utilized as an adjuvant therapy during IVF due to the theory that reducing microbial colonization within the upper genital tract will have positive effects on endometrial receptivity and thus pregnancy and live birth rates (16). The presence of infection has been suggested to negatively impact the likelihood of implantation and it has been demonstrated that antibiotics are effective in significantly reducing genital tract colonization (38). However, our results are in line with Brook et al’s (38) findings demonstrating that, even if a reduction to microbial colonization was achieved, the use of antibiotics failed to significantly alter the success of embryo transfers.

Being an androgen pre-hormone, DHEA is proposed to improve IVF outcomes by increasing intra-ovarian androgen concentrations and promoting folliculogenesis. Yet, despite its widespread use, there remains uncertainty surrounding the efficacy of DHEA. Exploration of its use in IVF has mostly been conducted in small studies with heterogeneous populations and thus associated biases (39). Nagel et al.’s aforementioned Cochrane paper (25) reviewed 12 RCT studies of DHEA and concluded that, when removing trials at high risk of bias, the use of DHEA demonstrated non-statistically significant findings and our analysis was consistent with this.

Of interest, significant positive effects were seen with the use of aspirin and steroids. On univariate analysis, dopamine antagonists also demonstrated the beneficial effects of increased pregnancy rates, live births and reduced pregnancy losses; however, these became non-significant after logistic regression.

Aspirin has been employed as an adjuvant to IVF treatment for its theoretical potential of improving uterine perfusion and thus endometrial receptivity (18). Aspirin is suggested to improve uterine blood flow by reducing platelet aggregation and vasoconstriction and lead to a more favourable endometrium for implantation (18, 40). Despite its theoretical potential, however, the use of aspirin as an adjunct to IVF remains controversial and results have been conflicting (41). Our analysis has detected a statistically significant improvement in live birth rates following embryo transfer. Our results align with previous results (42) showing aspirin’s positive impact on pregnancy outcomes; however, other studies have concluded there is no evidence for aspirin’s efficacy (40–41, 43). These five meta-analyses have been conducted investigating its use and four found a non-significant effect, believing the positive findings in small-scale studies are due to chance. Within these studies, the dosage of aspirin was typically 80–100 *mg* of daily aspirin, similar to our protocol, however there was wide variation in commencement and duration of aspirin use in the many studies included. The significant increase in live birth rates demonstrated by our data should be interpreted with caution within this context, and further investigation of aspirin’s benefits is recommended with randomized control trials. Future studies need to have adequate sample sizes to properly benefit from evidence-based practice as previous meta-analyses have been based on trials with limited numbers.

The use of steroids for improving IVF outcomes targets the immunological uterine environment in an attempt to alter cytokine and natural killer cell profiles for optimal implantation conditions (10). Intralipid was implicated to have similar theoretical potential, yet produced non-significant results within our study unlike our findings with the use of steroids. Our results with steroids use are the most promising within our analysis. Both a significant reduction in clinical pregnancy loss rates and significantly improved live birth rates were demonstrated in this study. Boomsma et al.’s Cochrane review (10) found no clear improvement of clinical outcomes with the administration of peri-implantation glucocorticoids overall but did find borderline statistical significant improvement in pregnancy rates for a subgroup of women undergoing IVF (rather than ICSI). Boomsma’s review included 13 trials assessing pregnancy rates with the use of glucocorticoids. Within these 13 trials, 894 patients received glucocorticoids out of the 1,759 patients involved. In our single study alone, we had 997 patients receiving steroid treatment and, again, whilst not randomized, our larger sample size perhaps provides the opportunity to demonstrate a difference that a meta-analysis of smaller randomized controlled trials would not. With our results indicating significant positive trends, there may be potential benefits for additional subsets of patients and this is worth exploring.

Literature surrounding the use of dopamine agonists as adjuvants to IVF treatment largely concerns their effectiveness in reducing ovarian hyperstimulation syndrome (OHSS). Dopamine agonists minimize VEGF-2 phosphorylation, and thus are proposed to reduce the extravasation of fluids causing OHSS (21). Our univariate analysis of dopamine agonist use found significantly increased pregnancy and live birth rates and this beneficial effect could be attributed to the use in high responder patients to avoid hyperstimulation syndrome. This hypothesis seems to be confirmed as on subsequent logistic regression, these positive effects became non-significant; however, direction of effect remained the same. Little research is available investigating any association between dopamine agonists and the success of embryo transfers and further exploration of potential benefit is needed.

For all adjuvant therapies, further investigation of their impact on the success of embryo transfers is needed with high-quality study designs. Limitations of our study include the possible selection bias of only including cycles in which a transfer occurred and not including those without a transfer. In addition, some therapies had a small sample size and therefore their results need to be interpreted with caution.

## Conclusion

Couples faced with unsuccessful IVF cycles may feel desperate to try anything to assist their chance of conception. This vulnerable population may eagerly utilize adjuvant therapies despite little evidence available to support their use. Many of the interventions investigated in this study fail to significantly demonstrate any effects on the success of embryo transfers and our analysis results show negative effects with the use of melatonin. Thus, continuous use of these adjuvants is not advisable until further high-quality trials with adequate sample sizes are performed. On the other hand, aspirin and steroids demonstrated promising, potentially beneficial outcomes, but additional exploration into the strength of their benefit is needed to guide evidence-based practice.
